# Mycoplasma genitalium Detection in Urogenital Specimens from Symptomatic and Asymptomatic Men and Women by Use of the cobas TV/MG Test

**DOI:** 10.1128/JCM.02124-19

**Published:** 2020-05-26

**Authors:** Barbara Van Der Pol, Ken B. Waites, Li Xiao, Stephanie N. Taylor, Arundhati Rao, Melinda Nye, Steven Chavoustie, Aaron Ermel, Clair Kaplan, David Eisenberg, Philip A. Chan, Leandro Mena, Sixto Pacheco, Smitha Krishnamurthy, Ruchika Mohan, Rasa Bertuzis, Chris L. McGowin, Rodney Arcenas, Elizabeth M. Marlowe

**Affiliations:** a University of Alabama at Birmingham School of Medicine, Birmingham, Alabama, USA; b Louisiana State University Health Sciences Center, New Orleans, Louisiana, USA; c Baylor Scott & White Health, Temple, Texas, USA; d Laboratory Corporation of America Holdings, Burlington, North Carolina, USA; e Healthcare Clinical Data, Inc., North Miami, Florida, USA; f Indiana University School of Medicine, Indianapolis, Indiana, USA; g Planned Parenthood of Southern New England, New Haven, Connecticut, USA; h Planned Parenthood of St. Louis Region and Southwest Missouri, St. Louis, Missouri, USA; i Brown University, Providence, Rhode Island, USA; j University of Mississippi Medical Center, Jackson, Mississippi, USA; k BioCollections Worldwide, Inc., Miami, Florida, USA; l Roche Molecular Systems, Inc., Pleasanton, California, USA; m Quest Diagnostics Infectious Disease, San Juan Capistrano, California, USA; Marquette University

**Keywords:** cobas TV/MG, *Mycoplasma genitalium*, molecular diagnostics, PCR, genital infection, genital disease

## Abstract

Mycoplasma genitalium (MG) infections are a growing concern within the field of sexually transmitted infections. However, diagnostic assays for M. genitalium have been limited in the United States. As most infections are asymptomatic, individuals can unknowingly pass the infection on, and the prevalence is likely to be underestimated. Diagnosis of M. genitalium infection is recommended using a nucleic acid test. This multicenter study assessed the performance of the cobas Trichomonas vaginalis (TV)/MG assay (cobas) for the detection of M. genitalium, using 22,150 urogenital specimens from both symptomatic and asymptomatic men and women collected at geographically diverse sites across the United States. The performance was compared to a reference standard of three laboratory-developed tests (LDTs). The specificity of the cobas assay for M. genitalium ranged from 96.0% to 99.8% across symptomatic and asymptomatic men and women. The sensitivities in female vaginal swabs and urine samples were 96.6% (95% confidence interval [CI], 88.5 to 99.1%) and 86.4% (95% CI, 75.5 to 93.0%), respectively. The sensitivities in male urine and meatal swab samples were 100% (95% CI, 94.0 to 100%) and 85.0% (95% CI, 73.9 to 91.9%), respectively. This study demonstrated that the cobas assay was highly sensitive and specific in all relevant clinical samples for the detection of M. genitalium.

## Abstract (precis)

Mycoplasma genitalium (MG) infections are a growing concern within the field of sexually transmitted infections. However, diagnostic assays for M. genitalium have been limited in the United States. As most infections are asymptomatic, individuals can unknowingly pass the infection on, and the prevalence is likely to be underestimated. Diagnosis of M. genitalium infection is recommended using a nucleic acid test. This multicenter study assessed the performance of the cobas Trichomonas vaginalis (TV)/MG assay (cobas) for the detection of M. genitalium, using 22,150 urogenital specimens from both symptomatic and asymptomatic men and women collected at geographically diverse sites across the United States.

## INTRODUCTION

Mycoplasma genitalium is a sexually transmitted infection (STI) which has been associated with urethritis, cervicitis, pelvic inflammatory disease, and male and female infertility in epidemiologic studies (1–8). The prevalence of M. genitalium infection varies depending on the geographical region, gender, and the presence of risk factors. In the general population, it is estimated to range from 1% to 2% (9–12), and in patients attending sexual health clinics, the estimates range from 3.3% to 38% (2, 13–18).

Many M. genitalium infections are asymptomatic, and, therefore, it is possible for individuals to unknowingly transmit the infection to their sexual partners (19–21). Asymptomatic infections can lead to pelvic inflammatory disease, which is associated with serious long-term sequelae, including ectopic pregnancy, infertility, and pelvic/abdominal pain (3, 22; www.cdc.gov/std/pid/stdfact-pid-detailed.htm). The extent to which these sequelae can be attributed to asymptomatic M. genitalium infections is unknown, in part due to a lack of sensitive diagnostic tools. M. genitalium is difficult to culture, typically requiring several weeks or months, meaning that, historically, M. genitalium infections were rarely diagnosed and it was difficult to estimate their prevalence (23; www.cdc.gov/std/tg2015/emerging.htm#myco). M. genitalium infections can now be rapidly detected using nucleic acid amplification tests (NAATs). Accurate detection of M. genitalium is important for the treatment of symptomatic infections, as many strains of M. genitalium have developed resistance to the empirical treatments for urethritis or cervicitis (3, 8, 13, 19, 24–27; www.cdc.gov/std/tg2015/emerging.htm#myco).

Despite its relatively high prevalence compared with other STIs such as gonorrhea, screening for M. genitalium infections in asymptomatic individuals is not recommended due to our limited understanding of the consequences of asymptomatic infection and the need for antimicrobial stewardship (i.e., not treating infections that may naturally clear without harm). Only targeted testing of symptomatic or high-risk individuals is recommended by the currently published guidelines for STI screening and treatment (3; www.cdc.gov/std/tg2015/emerging.htm#myco). In the United States, there are currently only two FDA-approved diagnostic tests for the detection of M. genitalium in urogenital specimens: the Aptima M. genitalium (APT MG) assay (Hologic, Inc., San Diego, CA) and the Roche cobas Trichomonas vaginalis (TV)/MG assay (cobas) (www.cdc.gov/std/tg2015/emerging.htm#myco; www.cdc.gov/std/stats17/gonorrhea.htm; https://diagnostics.roche.com/us/en/news-listing/2019/roche-receives-fda-clearance-to-expand-testing-menu-on-cobas-6800-8800-systems-for-sexually-transmitted-diseases.html; https://www.fda.gov/news-events/press-announcements/fda-permits-marketing-first-test-aid-diagnosis-sexually-transmitted-infection-known-mycoplasma). In 2015, the U.S. Centers for Disease Control and Prevention (CDC) recognized M. genitalium infections as an emerging concern and described the need for improvements in the diagnosis and treatment of these infections (www.cdc.gov/std/tg2015/emerging.htm#myco). The British Association for Sexual Health and HIV (BASHH) and the International Union Against Sexually Transmitted Infections (IUSTI) both recommend that symptomatic patients should be tested for M. genitalium infection using NAAT technologies (3, 28). The objective of this multicenter study was to evaluate the clinical performance of the cobas test for the detection of M. genitalium, using urogenital specimens from both symptomatic and asymptomatic men and women.

## MATERIALS AND METHODS

### Patient population and ethics.

This multicenter study enrolled 2,194 participants ≥14 years of age who reported sexual activity within the previous 6 months. Participants attending family planning, obstetrics and gynecology, and STI clinics were recruited from geographically diverse sites in the United States: Birmingham (Alabama), Indianapolis (Indiana), Jackson (Mississippi), Miami (Florida), New Haven (Connecticut), New Orleans (Louisiana), Oakland (California), Providence (Rhode Island), and St. Louis (Missouri) (Fig. S1).

Participants were classified as demonstrating signs of infection if they reported any of the following symptoms: dysuria, coital issues (pain, difficulty, or bleeding), pelvic pain, abnormal vaginal discharge, unusual vaginal odor pelvic, uterine or ovarian pain, penile discharge, testicular pain, scrotal pain, or swelling, itching, burning, and redness or soreness of the genitals.

Patients were ineligible if they had previously enrolled in the study; used antimicrobial agents active against M. genitalium (doxycycline, macrolides including azithromycin and erythromycin, or fluoroquinolones including ofloxacin, ciprofloxacin, and levofloxacin) within the 21 days prior to sample collection; used Replens (Church & Dwight, Co., Inc., Princeton, NJ), RepHresh Odor Eliminating Vaginal Gel, RepHresh Clean and Balance (Church & Dwight, Co., Inc., Princeton, NJ), or products containing metronidazole within 3 days prior to specimen collection; had undergone a full hysterectomy; or had a contraindication to the Papanicolaou test or cervical sampling.

This study was conducted in compliance with the International Conference on Harmonization of Technical Requirements for Pharmaceuticals for Human Use (ICH), Good Clinical Practice Guidelines (GCP), and applicable FDA regulations, and all participating subjects provided written informed consent. Institutional Review Board approval was obtained from each participating study site prior to the start of the study.

### Specimen collection.

Women provided specimens in the following order: first-catch urine (FCU), vaginal swabs, an endocervical swab in cobas PCR media, and a cervical specimen in PreservCyt solution obtained with a spatula, cytobrush, or broom. Participants were randomized to either self-obtained or clinician obtained for collection of vaginal swabs used in the cobas assay.

Participants within the self-collected arm had their self-collected vaginal swab collected first, and the remaining swabs were clinician collected. In the clinician-collected arm, all vaginal swabs were clinician collected. Following collection, the clinician transferred the swabs to the relevant transport media, as per the respective laboratory’s standard operating procedures, for the validated APT MG assay (Hologic, San Diego, CA) and two M. genitalium laboratory-developed tests (LDTs) (29–31). Participants within the clinician-collected arm had an additional clinician-collected specimen for use with the cobas test. Both the endocervical swab and the liquid-based cytology (LBC) sample were collected for assessment with the cobas assay only.

Men first provided meatal swabs (self- or clinician collected) for use with the cobas test, followed by an FCU sample. The FCU sample was aliquoted into the manufacturer’s collection device for use with APT MG, the two other M. genitalium LDTs, and the cobas assay.

### Sample testing.

The cobas assay was tested on either the cobas 6800 or 8800 system (detection of M. genitalium with the cobas assay is FDA cleared for female urine, self- and clinician-collected vaginal swabs, endocervical swabs, male urine, and male meatal swabs only). Specimens from each subject were tested using the cobas assay at a single test site. Samples for comparator methods were tested at sites based on the availability of the comparator instrument system and method. Samples were coded to ensure they were anonymized and to reduce bias. Testing was performed with each method according to the validated laboratory procedure (for the three LDTs). One of the M. genitalium LDTs was a real-time PCR assay that targeted the *mgpA* gene of M. genitalium (29, 30). The other M. genitalium LDT was a quantitative PCR designed to target the 23S rRNA gene of M. genitalium (31). The APT MG assay detects the 16S rRNA of M. genitalium.

### Patient infected status.

The patient infected status (PIS) was determined from vaginal swabs (women) and FCU (men) assayed in two M. genitalium laboratory-developed NAATs and the APT MG assay. If a participant had two or more positive results, the PIS was “positive,” and at least two negative results defined the “not infected” classification. Any other combinations of valid results with invalid results were considered “indeterminate.” Performance estimates for all sample types were based on comparison to these PIS classifications.

### Data analysis and interpretation of results.

Test results for each assay were interpreted according to the testing laboratory’s standard operating procedures (SOP) and validation for their respective M. genitalium assay. Results were deemed invalid if there were protocol deviations, incidents, or if the data were generated during troubleshooting of the instrument or assays. All data analyses were performed using SAS/STAT software (32).

The clinical performance of the cobas test for the detection of M. genitalium was evaluated by comparing test results to the PIS. The sensitivity, specificity, positive predictive value (PPV), and negative predictive value (NPV) were calculated overall, for each gender, and by specimen type and symptom status and were compared with the infected status. The two-sided 95% confidence intervals (CIs) were provided for the estimates of sensitivity, specificity, PPV, and NPV. Significance was defined using *Z*-test analysis with alpha = 0.05.

## RESULTS

### Subject disposition.

Of the 2,194 participants enrolled in the study, a total of 2,154 were considered eligible, and 2,150 were evaluated (1,104 female and 1,046 male) for the assessment of M. genitalium infection (Table 1). Evaluable urine samples were available from 1,099 female and 1,045 male participants. Clinician-collected and self-collected vaginal swabs were available in 551 and 550 participants, respectively. Clinician-collected and self-collected penile meatal swabs were available from 516 and 522 participants, respectively. In total, 28 specimens were excluded from the analysis: 5 female urine, 2 clinician-collected vaginal swabs, 1 self-collected vaginal swab, 6 PreservCyt, 5 endocervical swabs, 1 male urine, 2 clinician-collected meatal swabs, 2 self-collected meatal swabs, and 4 meatal swabs without collection information.

**TABLE 1:** Baseline demographics and characteristics

Characteristic	Value(s)
Total (*n*)	2,150
Male age, yrs (mean ± SD)	37.6 ± 13.6
Female age, yrs (mean ± SD)	34.2 ± 11.7
Male (*n* [%])	1,046 (48.7)
Female (*n* [%])	1,104 (51.3)
American Indian/Alaskan Native (*n* [%])	3 (0.1)
Asian (*n* [%])	13 (0.6)
Black/African American (*n* [%])	1,501 (69.8)
Native Hawaiian/Pacific Islander (*n* [%])	5 (0.2)
White (*n* [%])	553 (25.7)
Multiple/other (*n* [%])	55 (2.6)
Not reported (*n* [%])	20 (0.9)
Symptomatic (*n* [%])	984 (45.8)
Asymptomatic (*n* [%])	1,166 (54.2)
Pregnant (female only) (*n* [%])	3 (0.3)
No. (%) of patients at a(n):	
Family planning clinic	525 (24.4)
Obstetrics/gynecology clinic	273 (12.7)
STI clinic	758 (35.2)
Family planning/STI clinic	594 (27.6)

### Assay performance for the detection of M. genitalium.

**FIG 1:**
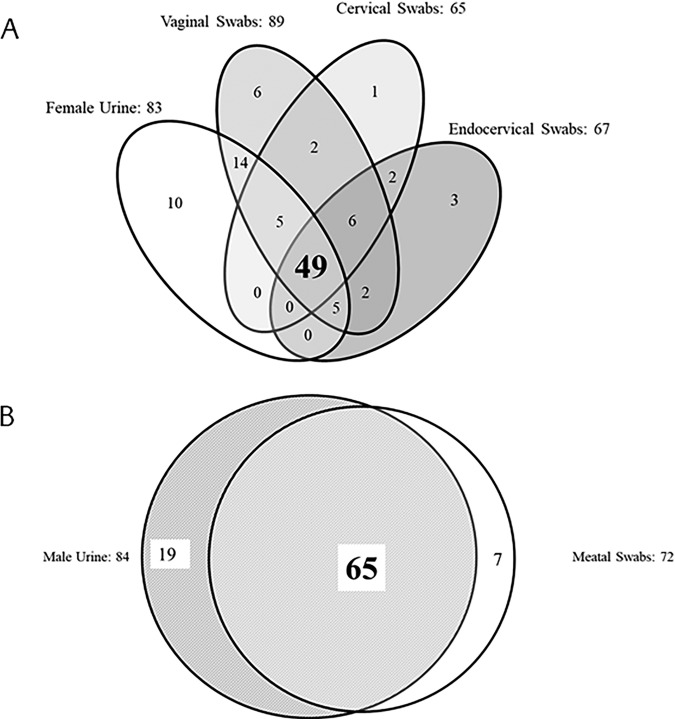
Venn diagrams comparing M. genitalium-positive female urogenital samples (A) and male urogenital samples (B). These data show exclusively cobas M. genitalium-positive results, as each sample type was not tested by all comparator assays.

In total, 59 women and 60 men were considered infected as determined by PIS analysis. Of these infected participants, 67.8% of women and 51.7% of men reported symptoms. The sensitivity, specificity, PPV, and NPV of cobas for the detection of M. genitalium are shown in Table 2. The overall sensitivity of the cobas test for the detection of M. genitalium in women was highest in vaginal swab samples (96.6% [95% CI, 88.5 to 99.1]; clinician and self-collected combined). The overall sensitivity of the test for female urine, PreservCyt samples, and endocervical samples ranged from 83.1% to 86.4% (Table 2). The overall sensitivity of cobas for M. genitalium in male urine samples and meatal swab samples was 100% (95% CI, 94.0 to 100%) and 85.0% (95% CI, 73.9 to 91.9%), respectively. There were no statistically significant sensitivity differences between the clinician- and self-collected vaginal swabs (96.3% versus 96.9%, respectively; *P* > 0.99) and meatal swabs (83.9% versus 86.2%, respectively; *P* > 0.99) as determined by the *Z*-test analyses. Additional *Z*-test analyses similarly showed no statistically significant specificity differences between the clinician- and self-collected vaginal swabs (96.8% versus 97.3%, respectively; *P* = 0.63) and meatal swabs (97.5% versus 98.2%, respectively; *P* = 0.74). Venn diagrams comparing cobas M. genitalium positivity across all tests, regardless of PIS, in female urine, male urine, vaginal, and meatal swab samples are shown in Fig. 1. The specificity of the cobas assay for M. genitalium ranged from 96.0 to 99.8% across male and female symptomatic and asymptomatic samples (Table 2).

**TABLE 2:** Clinical performance compared with PIS by gender, sample type, and symptom status

Sample type	Total	Sensitivity % (no. of true positives detected by cobas MG/total no. of true positives)	95% CI	Specificity % (no. of true negative samples identified/total no. of true negatives)	95% CI	Prevalence (%)	PPV (%)	NPV (%)
Female participants								
Urine								
Symptomatic	636	85.0 (34/40)	70.9–92.9	96.0 (572/596)	94.1–97.3	6.3	58.6	99.0
Asymptomatic	463	89.5 (17/19)	68.6–97.1	98.4 (437/444)	96.8–99.2	4.1	70.8	99.5
Overall	1,099	86.4 (51/59)	75.5–93.0	97.0 (1,009/1,040)	95.8–97.9	5.4	62.2	99.2
Vaginal swab (both clinician and self-collected)								
Symptomatic	639	97.5 (39/40)	87.1–99.6	96.3 (577/599)	94.5–97.6	6.3	63.9	99.8
Asymptomatic	462	94.7 (18/19)	75.4–99.1	98.0 (434/443)	96.2–98.9	4.1	66.7	99.8
Overall	1,101	96.6 (57/59)	88.5–99.1	97.0 (1,011/1,042)	95.8–97.9	5.4	64.8	99.8
PreservCyt samples								
Symptomatic	638	80.0 (32/40)	65.2–89.5	97.8 (585/598)	96.3–98.7	6.3	71.1	98.7
Asymptomatic	460	94.7 (18/19)	75.4–99.1	99.8 (440/441)	98.7–100	4.1	94.7	99.8
Overall	1,098	84.7 (50/59)	73.5–91.8	98.7 (1,025/1,039)	97.8–99.2	5.4	78.1	99.1
Endocervical swab								
Symptomatic	637	85.0 (34/40)	70.9–92.9	97.7 (583/597)	96.1–98.6	6.3	70.8	99.0
Asymptomatic	462	78.9 (15/19)	56.7–91.5	99.3 (440/443)	98.0–99.8	4.1	83.3	99.1
Overall	1,099	83.1 (49/59)	71.5–90.5	98.4 (1,023/1,040)	97.4–99.0	5.4	74.2	99.0
Male participants								
Urine								
Symptomatic	343	100 (31/31)	89.0–100	96.8 (302/312)	94.2–98.2	9.0	75.6	100
Asymptomatic	702	100 (29/29)	88.3–100	97.9 (659/673)	96.5–98.8	4.1	67.4	100
Overall	1,045	100 (60/60)	94.0–100	97.6 (961/985)	96.4–98.4	5.7	71.4	100
Meatal swab (both clinician- and self-collected)								
Symptomatic	343	90.3 (28/31)	75.1–96.7	96.5 (301/312)	93.8–98.0	9.0	71.8	99.0
Asymptomatic	695	79.3 (23/29)	61.6–90.2	98.5 (656/666)	97.3–99.2	4.2	69.7	99.1
Overall	1,038	85 (51/60)	73.9–91.9	97.9 (957/978)	96.7–98.6	5.8	70.8	99.1

Based on PIS, M. genitalium prevalence was higher in symptomatic than asymptomatic patients, and the overall prevalence ranged from 5.4% to 5.8% across male and female specimens (Table 2). The PPV of the cobas for detection of M. genitalium was 58.6 to 94.7%, and the NPV was 98.7 to 100% across all specimen types evaluated. Additional analyses of M. genitalium (regardless of PIS) prevalence by age, gender, sample type, and study site are provided in Table S1 and S2.

## DISCUSSION

This multicenter study evaluated the clinical performance of the cobas test for the detection of M. genitalium in urine and genital swab samples from men and women. Male urine and female vaginal swab samples had the highest sensitivity and specificity for the detection of M. genitalium in this analysis. The evidence supporting optimal specimen collection for M. genitalium detection in urogenital specimens is evolving. Observed differences among specimen types may be associated with pathogenesis and anatomical location (33, 34). The prevalence of M. genitalium varied among female specimens (Table S2). However, the differences between specimen types for men were not significant. The only statistically significant differences among female samples were between cervical (PreservCyt) and endocervical swabs, which were significantly less sensitive compared with vaginal swabs (Table 2) (*P* < 0.0001).

The cobas test for the detection of M. genitalium had similar performance when assessed in both self-collected and clinician-collected vaginal or meatal swabs. This is important, as self-collection allows patients who are not comfortable with visiting a clinic or clinician collection access to effective testing. Across the STI testing field, self-testing has provided increased access to testing for patients who otherwise may not have received testing and is considered to have similar performance to testing with clinician-collected samples (35–38).

Specificity is important to ensure a patient is truly positive for the test infection. This is particularly important when introducing new NAATs to become the standard of care when gold-standard culture tests have historically been unavailable. The specificity of the cobas TV/MG test for the detection of M. genitalium was high regardless of the sample type or symptom status (Table 2), indicating the ability to perform well in different patient populations. In the absence of a reliable gold-standard test for the detection of M. genitalium, the first FDA-approved assay (Hologic Aptima) was validated by comparison to three alternate thermomechanical analysis (TMA) LDTs (18, 39). Here, we provide a similar evidence base for the cobas assay, allowing comparison with three validated LDTs (two PCR and one TMA-based method). Table 3 shows the head-to-head comparisons of cobas with the individual M. genitalium LDT NAATs for the U.S. prospective clinical study and highlights the variability that may be observed with different laboratories using validated LDTs for diagnosis of a suspected M. genitalium infection.

**TABLE 3:** Agreement of cobas for M. genitalium with each NAAT

Test or statistic	No. with NAAT1 test result of:		Total	No. with NAAT2 test result of:		Total	No. with NAAT3 test result of:		Total
	M. genitalium positive^*a*^	M. genitalium-negative NAAT1		M. genitalium positive^*b*^	M. genitalium negative		M. genitalium positive^*c*^	M. genitalium-negative NAAT3	
Vaginal swabs									
M. genitalium positive	36	52	88	55	33	88	88	0	88
M. genitalium negative	13	999	1,012	10	1,002	1,012	26	986	1,012
Total	49	1,051	1,100	65	1,035	1,100	114	986	1,100
			
PPA (% [95% CI])	73.5 (59.7–83.8)			84.6 (73.9–91.4)			77.2 (68.7–83.9)		
NPA (% [95% CI])	95.1 (93.6–96.2%			96.8 (95.6–97.7)			100 (99.6–100)		
OPA (% [95% CI])^*d*^	94.1 (92.5–95.3)			96.1 (94.8–97.1)			97.6 (96.6–98.4)		
Male urine samples									
M. genitalium positive	57	27	84	52	32	84	79	5	84
M. genitalium negative	12	943	955	5	950	955	3	952	955
Total	69	970	1,039	57	982	1,039	82	957	1,039
			
PPA (% [95% CI])	82.6 (72.0–89.8)			91.2 (81.1–96.2)			96.3 (89.8–98.7)		
NPA (% [95% CI])	97.2 (96.0–98.1)			96.7 (95.4–97.7)			99.5 (98.8–99.8)		
OPA (% [95% CI])	96.2 (94.9–97.2)			96.4 (95.1–97.4)			99.2 (98.5–99.6)		

a NAAT1 represents LDT 1 (targets the *mgbA* gene).

b NAAT2 represents LDT 2 (targets 23S rRNA).

c NAAT3 represents LDT3 (targets 16S rRNA).

d OPA, overall percentage agreement.

This prospective clinical study assessed the performance of the cobas assay for detecting M. genitalium among both symptomatic and asymptomatic patients. Current European and BASHH guidelines recommend testing of symptomatic individuals, but it is left to the discretion of the health care provider whether testing is warranted in those who are asymptomatic. In agreement with this study, the European and BASHH guidelines currently recommend that FCU samples in male participants and female vaginal swabs are the most sensitive sample types (3, 28). This study did not include anorectal samples in the evaluation since such studies should be conducted in more specialized clinical settings that provide services to men who have sex with men. This is an important area for future assay evaluations.

In this multicenter clinical study, the cobas assay had high sensitivity and specificity for the detection of M. genitalium in both male and female sample types, regardless of symptom status. This study provides evidence of a fully validated, high-throughput PCR assay for the detection of M. genitalium. Diagnostic solutions that include resistance markers in addition to the detection of the organism may be necessary in the near future. A useful aspect of the cobas 6800/8800 system is that LDTs can be rapidly developed and implemented on this platform, as reflex test options for M. genitalium-positive specimens are required (40).

## Supplementary Material

Supplemental file 1
