# Supplementary material for: Mycoplasma genitalium Detection in Urogenital Specimens from Symptomatic and Asymptomatic Men and Women by Use of the cobas TV/MG Test
Source: J Clin Microbiol. 2020 May 26;58(6):e02124-19. doi: 10.1128/JCM.02124-19 (PMC7269414; doi:10.1128/JCM.02124-19)
Supplement: Supplemental file 1 [file JCM.02124-19-s0001.pdf]

Supplemental Table 1. MG prevalence using the cobas assay, by gender and age.

Age Category	MG Total Number of cobas Evaluable Samples	MG Total Number of cobas Positive Samples	MG Prevalence (%)
Female			
Between 14 and 20 years	267	26	9.7% (26/267)
Between 21 and 30 years	1,884	187	9.9% (187/1885)
Between 31 and 40 years	1,077	56	5.2% (56/1076)
Between 41 and 50 years	559	13	2.3% (13/559)
Between 51 and 60 years	533	22	4.1% (22/532)
Between 61 and 70 years	92	22	23.9% (22/92)
Total	4,412	304	6.9% (304/4411)
Male			
Between 14 and 20 years	98	8	8.2% (8/98)
Between 21 and 30 years	719	86	12.0% (86/719)
Between 31 and 40 years	518	39	7.5% (39/518)
Between 41 and 50 years	254	9	3.5% (9/254)
Between 51 and 60 years	350	10	2.9% (10/350)
Between 61 and 70 years	132	4	3.0% (4/132)

Age Category	MG Total Number of cobas Evaluable Samples	MG Total Number of cobas Positive Samples	MG Prevalence (%)
Between 71 and 80 years	12	4	33.3% (4/12)
Total	2,083	156	7.5% (156/2083)

Data are cobas MG positive results, regardless of the PIS.

MG, *Mycoplasma genitalium*; PIS, patient infected status.

Supplemental Table 2. MG prevalence using cobas® MG, by gender and sample type.

Sample Type	MG Total Number of cobas-Evaluable Samples	MG Total Number of cobas Positive Samples	MG Prevalence (%)
Female samples			
Urine	1,104	83	7.5% (83/1103)
Vaginal Swab	1,104	89	8.1% (89/1104)
Cervical Swab	1,102	65	5.9% (65/1102)
Endocervical Swab	1,102	67	6.1% (67/1102)
Total	4,412	304	6.9% (304/4411)
Male samples			
Urine	1,045	84	8.0% (84/1045)
Meatal Swab	1,038	72	6.9% (72/1038)
Total	2,083	156	7.5% (156/2083)

MG, *Mycoplasma genitalium*.

Supplemental Figure 1. MG prevalence using the cobas assay for MG, by gender and study site.

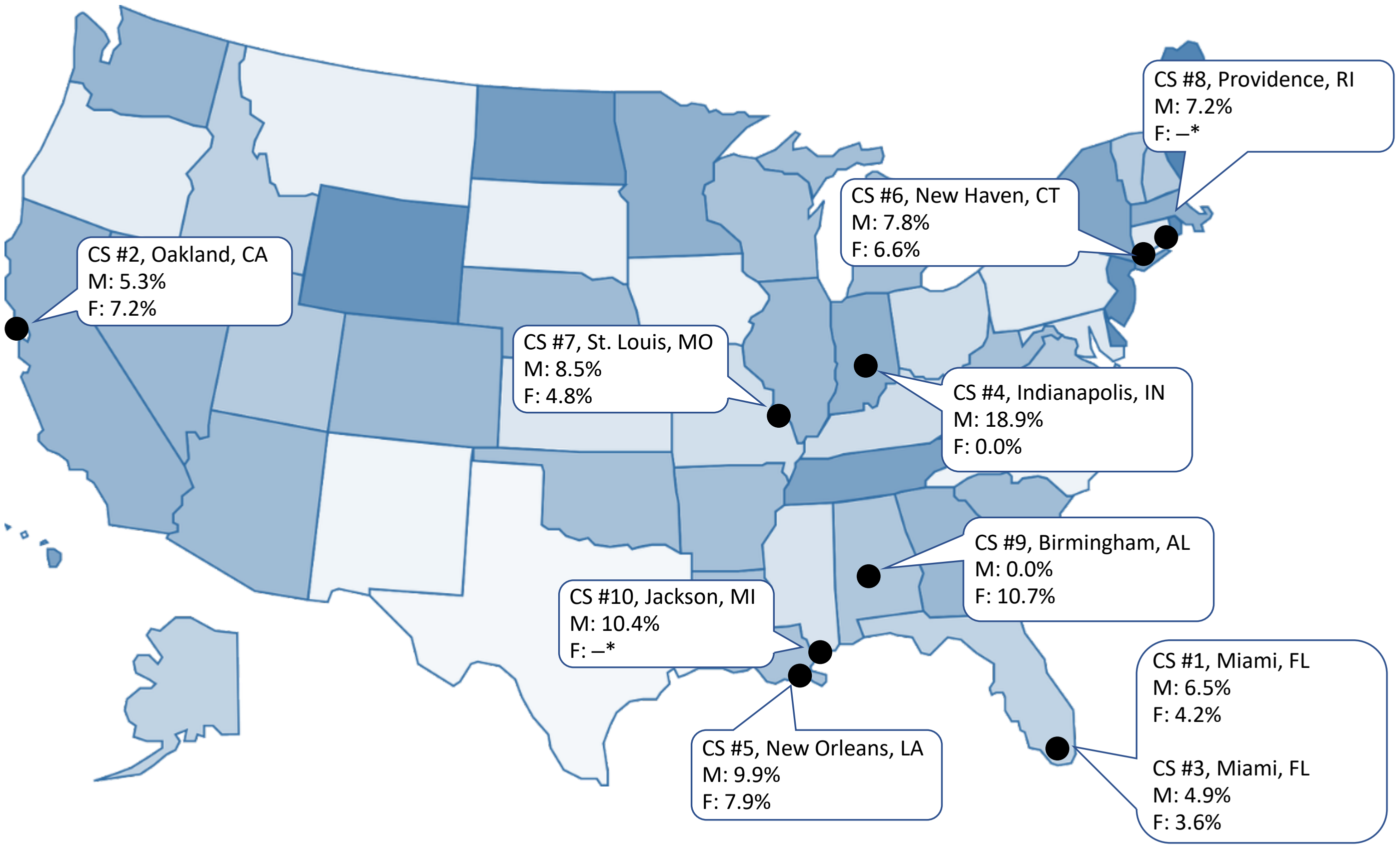

*No female subjects enrolled.

CS, collection site; F, female; M, male; MG, *Mycoplasma genitalium*
